# Metal 3D printing: Patent law, trade secrets, and additive manufacturing

**DOI:** 10.3389/frma.2022.958761

**Published:** 2022-09-02

**Authors:** Matthew Rimmer

**Affiliations:** Faculty of Business and Law, Queensland University of Technology, Brisbane, QLD, Australia

**Keywords:** 3D printing (3DP), intellectual property (IP), patent law, trade secrets, additive manufacturing

## Abstract

There has been significant investment in research and development in respect of metal 3D printing in the United States (as well as a number of other jurisdictions). There has been growing conflict over the ownership of intellectual property in respect of metal 3D printing (involving not only patents but also trade secrets and confidential information, as well as contract law and unfair competition). In 2018, Desktop Metal Inc. launched litigation against Markforged Inc. and Matiu Parangi in relation to intellectual property and metal 3D printing in the United States. As well as complaints of patent infringement, Desktop Metal Inc. has alleged that the defendants had engaged in acts of trade secret misappropriation, unfair and deceptive business practices, and breach of contract. Markforged Inc. made various counter-claims of its own. In July 2018, a Federal Jury found that Markforged Inc. did not infringe two patents held by its rival Desktop Metal Inc. Claims of further violations of trade secrets and contract law were also considered. In the end, the dispute was settled, with neither party obtaining an advantage in the litigation. There was further conflict over whether the terms of the settlement in respect of non-disparagement were honored. The parties have also faced further intellectual property conflict. In 2021, Continuous Composites has filed a patent infringement lawsuit against Markforged Inc. In 2021, Desktop Metal Inc. brought legal action against SprintRay in Germany. Drawing upon this case study, this paper considers whether metal 3D printing will disrupt patent law, policy, and practice. It also explores the tension between the use of trade secrets in commercial 3D printing (such as in metal 3D Printing), and the open source ethos of the Maker Movement. This paper considers the larger implications of this intellectual property dispute over metal 3D printing for scarcity, regulation, and the abundance society. It also explores the innovation policies of the Biden administration in respect of advanced manufacturing—with a focus upon metal 3D printing and additive manufacturing.

## Introduction

There has been a significant concentration of patents in the field of 3D printing, and a diversification of subject matter in terms of the patent claims. One of the emerging trends in patent landscapes has been the rise of patents in respect of metal 3D printing. There has been significant investment in research and development in respect of metal 3D printing in the United States, as well as a number of other jurisdictions, including Canada, the European Union, and Australia. There has been significant investment in such forms of advanced manufacturing by middle tier countries, like China, as well (Birtchnell et al., 2018).

There has been increasing patent analytic work in respect of 3D Printing. The World Intellectual Property Organization (2015) conducted a survey of patent landscapes of various breakthrough technologies—including 3D printing. There has been a steady growth in patent applications by private companies in respect of 3D printing and other breakthrough technologies. There has also been significant patent activity by public research institutions (Rimmer, 2020a). The European Commission (2016) has focused on 3D printing and additive manufacturing as a priority in terms of its innovation policies. The European Parliament (2018) has issued a report on the policy challenges involved with the regulation of 3D printing. The European Patent Office (2020a,b) has been engaging in empirical research in respect of patent information in relation to 3D printing. The United Kingdom Intellectual Property Office has commissioned some specialist studies of intellectual property and 3D Printing (Birtchnell et al., 2018). IP Australia (2017) has studied, more broadly, the patent landscapes in respect of advanced manufacturing.

Analysts have conducted patent landscapes in relation to the specific sub-field of metal 3D printing. SmarTech Publishing (2018) published a qualitative analysis of metal 3D printing patents. Its study involved a database of almost 2,300 patents. SmarTech Publishing (2018) observed: “While patent litigation is fairly minimal at this point in time, SmarTech sees that situation changing as the metal 3D printing market continues to grow.” SmarTech Publishing (2018) predicted: “The firm expects to see greater activity in firms looking to protect market position or invalidate existing patents.” Moreover, SmarTech Publishing (2018) commented: “Expect more efforts to drive licensing relationships as well.” IFI Claims Patent Services created a 20,000+ database of patents classified under the category of additive manufacturing (Everett, 2021). The field of 3D printing patents was the ninth fastest growing field of technology in 2020 (Everett, 2021).

While initially there was copyright litigation over 3D printing (Rimmer, 2017), there has increasingly been disputes over patent law and trade secrets in the field of 3D Printing. Desai and Magliocca (2014) were prescient in predicting a rise in patent infringement disputes in respect of 3D printing and advanced manufacturing. There has been major commercial interest in the field of metal 3D printing, and significant conflict over the ownership of intellectual property (covering not only patents but also trade secrets). In 2018, Desktop Metal Inc. launched litigation against Markforged Inc. and Matiu Parangi in relation to intellectual property and metal 3D printing. As well as complaints of patent infringement, Desktop Metal Inc. has alleged that the defendants had engaged in acts of trade secret misappropriation, unfair and deceptive business practices, and breach of contract. In July 2018, a Federal Jury found that Markforged Inc. did not infringe two patents held by its rival Desktop Metal Inc. Claims of further violations of trade secrets and contract law were also considered. In the end, the dispute was settled, with neither party obtaining an advantage in the litigation. There was further conflict over whether the terms of the settlement in respect of non-disparagement were honored.

Since the conclusion of this dispute, the parties have also faced further intellectual property conflict with other parties. In 2021, Continuous Composites Inc. has filed a patent infringement lawsuit against Markforged Inc. This conflict is still in progress in the courts. Likewise, in 2021, Desktop Metal Inc. has brought legal action against SprintRay in Germany.

As recognized by Lemley (2015), the field of 3D printing poses fundamental challenges for intellectual property law, with the potential of technological abundance disrupting the artificial scarcity created by legal devices. Drawing upon this case study of the dispute between Desktop Metal Inc. and Markforged Inc., this paper considers whether metal 3D printing will disrupt patent law, policy, and practice. It also explores the tension between the use of trade secrets in commercial 3D printing (such as in metal 3D Printing), and the open source ethos of the Maker Movement. This paper provides a case study of the intellectual property conflict between Desktop Metal Inc. and Markforged Inc. over metal 3D printing. Part 1 compares and contrasts the two companies—and discusses their approach to intellectual property management and commercialization. Part 2 explores the patent dispute between Desktop Metal Inc. and Markforged Inc. It considers the mixed outcome of patent trial. While Desktop Metal Inc.'s patents were held to be valid, it was found that Markforged Inc. had not infringed any of those patents. Part 3 focuses on the competing claims of the parties in relation to trade secrets, consumer law, and contract law. Part 4 outlines the short-lived trial in respect of trade secrets and related matters, and details the confidential settlement between Desktop Metal Inc. and Markforged Inc. It considers the action over the alleged breach of a Non-Disparagement clause in the settlement. Part 5 notes further litigation—involving Continuous Composites Inc. bringing a patent infringement action against Markforged Inc., and Desktop Metal Inc. suing SprintRay for patent infringement in Germany. The conclusion explores the ramifications of the dispute for the larger theoretical debate over intellectual property and artificial scarcity; regulation; and the abundance society. It is predicted that there will be intense legal competition over the future of metal 3D printing, and the relative scarcity and abundance of the technology.

## The parties

Patent landscapes have highlighted that there are particular regions around the world, which have concentrated expertise in 3D printing and additive manufacturing.

The Boston area has long been an epicenter of innovation in new technologies—particularly with spin-offs from M.I.T. and Harvard University. Boston has a particularly luminous reputation for innovation in respect of 3D printing and additive manufacturing. Boston certainly could be considered to be a “Maker City” (Hirshberg et al., 2016; Rimmer, 2021). Of particular note, Gershenfeld (2005) has been a pioneer at M.I.T. in developing Fab Labs and personal fabrication. The Fab Lab movement has evolved into a larger digital revolution (Gershenfeld et al., 2017).

Sher (2018) commented that “it now appears clear that the city that is most closely associated with the American Revolution is rapidly becoming the center of another revolution: the additive manufacturing revolution.” He noted: “The entire FabLab community—of which 3D printing is a key element although not the only one—originated at MIT thanks to the work by Neil Gerhsenfeld and his Center for Bits and Atoms” (Sher, 2018). Sher (2018) commented: “Other MIT projects have made intensive use of 3D printing for robotics development, with the MIT CSAIL center working on everything from design software to self-assembling structures and new materials.” He also reflected: “Harvard's most high-profile 3D printing related initiatives are very much focused on bioprinting and biocompatible applications thanks to the work of the Jennifer Lewis Lab at the Wyss Institute for Bioengineering” (Sher, 2018). Sher highlighted a number of Boston-based companies—including Formlabs, Rize, Wyss, Onshape, Dassault Systems, Desktop Metal, and Markforged.

This patent dispute involves two of the flagship metal 3d printing companies in Boston—Desktop Metal Inc. and Markforged Inc.

### Desktop metal inc.

In its lawsuit against Markforged Inc. (Jackson, 2018a), Desktop Metal Inc. presents itself as a paragon of the metal 3D printing industry:

Desktop Metal, based in Burlington, Massachusetts, is accelerating the transformation of manufacturing with end-to-end metal 3D printing solutions. Founded in 2015 by leaders in advanced manufacturing, metallurgy, and robotics, the company is addressing the unmet challenges of speed, cost, and quality to make metal 3D printing an essential tool for engineers and manufacturers around the world. Desktop Metal is reinventing the way engineering and manufacturing teams produce metal parts—from prototyping through mass production (Desktop Metal., Inc., Complaint).

The company boasted: “In 2017, Desktop Metal was named to MIT Technology Review's list of 50 Smartest Companies and its products were recognized as among the most important innovations in engineering in Popular Science's “2017 Best of What's New” (Desktop Metal., Inc., Complaint). The company commented: “Since its inception in October 2015, Desktop Metal has raised a total of $277 million in financing, with its Series D marking the largest round ever for an additive manufacturing company at the time” (Desktop Metal., Inc., Complaint).

The company elaborates upon its raison d'etre: “Desktop Metal was founded in 2015 to address a problem—how to make metal 3D printing accessible for engineering teams” (Desktop Metal., Inc., Complaint). The company noted: “Metal 3D printing had failed to meet modern manufacturing needs due to high costs, slow processes, and hazardous and hazardous materials” (Desktop Metal., Inc., Complaint). Desktop Metal envisaged: “With a team of some of the world's leading experts in materials science, engineering, and innovation, Desktop Metal eliminated these barriers by developing metal 3D printing systems that can safely produce complex, strong metal parts at scale” (Desktop Metal., Inc., Complaint). The company maintained: “Desktop Metal's technology offers a new way for the manufacturing industry to be smarter, faster, and more cost effective with metal (Desktop Metal., Inc., Complaint).

Desktop Metal also seeks to paint a portrait of its principal figures in its lawsuit. The company noted: “Desktop Metal's CEO, Ric Fulop, has spent more than 25 years as an entrepreneur and high technology investor” (Desktop Metal., Inc., Complaint). The company observed: “In addition to CEO Ric Fulop, members of the founding team include some of the most forward-thinking innovators in the industry: Jonah Myerberg, Chief Technology Officer and a leader in materials engineering; Ely Sachs, MIT professor and early pioneer of 3D printing, inventor of binder jet printing; Yet-Ming Chiang, MIT professor and one of the world's top materials scientists; Christopher Schuh, Chairman of the MIT Dept. of Materials Science & Engineering and one of the world's leading metallurgists; A. John Hart, MIT professor and expert in manufacturing and machine design; and Rick Chin, VP of Software, who was one of the early team members of SolidWorks and previously founder of Xpress 3D (acquired by Stratasys, Ltd.)” (Desktop Metal., Inc., Complaint).

In its annual report, Desktop Metal Inc. highlights the importance that it places upon intellectual property. The company comments:

Our ability to drive innovation in the additive manufacturing market depends in part upon our ability to protect our core technology and intellectual property. We attempt to protect our intellectual property rights, both in the United States and abroad, through a combination of patent, trademark, copyright and trade secret laws, as well as non-disclosure and invention assignment agreements with our consultants and employees and through non-disclosure agreements with our vendors and business partners. Unpatented research, development, know-how and engineering skills make an important contribution to our business, but we pursue patent protection when we believe it is possible and consistent with our overall strategy for safeguarding intellectual property (Desktop Metal Inc, 2021, p. 16).

Accordingly, the company relies upon a combination of forms of intellectual property protection—primarily, patent law, and secondarily, through trade mark law, copyright law, and trade secrets law.

The company notes that it faces a number of risks related to intellectual property. In particular, Desktop Metal Inc. notes that it “may incur substantial costs enforcing and defending our intellectual property rights” (Desktop Metal Inc, 2021, p. 35). The company elaborates that the protection and enforcement of intellectual property rights is expensive and costly:

We may incur substantial expense and costs in protecting, enforcing, and defending our intellectual property rights against third parties. Intellectual property disputes may be costly and can be disruptive to our business operations by diverting attention and energies of management and key technical personnel and by increasing our costs of doing business. Third-party intellectual property claims asserted against us could subject us to significant liabilities, require us to enter into royalty and licensing arrangements on unfavorable terms, prevent us from assembling or licensing certain of our products, subject us to injunctions restricting our sale of products, cause severe disruptions to our operations or the marketplaces in which we compete or require us to satisfy indemnification commitments with our customers, including contractual provisions under various license arrangements. In addition, we may incur significant costs in acquiring the necessary third-party intellectual property rights for use in our products. Any of these could have an adverse effect on our business and financial condition (Desktop Metal Inc, 2021, p. 35).

The company also notes that “Third-party lawsuits and assertions to which we are subject alleging our infringement of patents, trade secrets or other intellectual property rights may have a significant adverse effect on our financial condition” (Desktop Metal Inc, 2021, p. 35). Desktop Metal also comments: “If we are unable to adequately protect or enforce our intellectual property rights, such information may be used by others to compete against us, in particular in developing consumables that could be used with our printing systems in place of our proprietary consumables” (Desktop Metal Inc, 2021, p. 35).

Desktop Metal Inc. has obtained registration under trade mark law for key terms such as “Desktop Metal,” “DM,” “Live Parts,” “Bound Metal Deposition,” “Studio System,” “BMD,” “Fabricate,” “Fab Flow,” and “Fiber.” It is worth noting in passing that Desktop Metal Inc. have also been involved in the US Trademark Trial and Appeal Board Inquiry system in respect of applications regarding trade marks for “Production System,” “Ceramic Release Layer,” “Separable Supports,” “Single Pass Jetting,” and “Bound Metal Deposition.”

The company also heavily relies upon trade secrets protection.

The approach of Desktop Metal Inc. to the management and commercialization of its intellectual property—with a combination of patent protection, trade mark protection, and trade secrets protection—is quite a marked contrast to the open source ethos of the Maker Movement.

In a review of the company and its technology, Rotman (2017) observed: “If it succeeds, Desktop Metal will help solve a daunting challenge that has eluded developers of 3-D printing for more than three decades, severely limiting the technology's impact.” Rotman (2017) noted: “Though it is possible to 3D-print metals, doing so is difficult and pricey.” Rotman (2017) commented: “Desktop Metal thinks its machines will give designers and manufacturers a practical and affordable way to print metal parts.” Rotman (2017) observed: “Having an affordable and fast way to print metal parts would be an important step in making this vision a reality.”

### Markforged inc.

In its Form 10-Q to the United States Securities and Exchange Commission, the Markforged Holding Corporation (2021) explains its approach to intellectual property management.

The Markforged Holding Corporation (2021, p. 1) observed that metal 3D printing was a dynamic field: “The additive manufacturing industry in which we operate is characterized by rapid technological change, which requires us to continue to develop new products and innovations to meet constantly evolving customer demands and which could adversely affect market adoption of our products.” The Markforged Holding Corporation (2021, p. 31) highlighted that the additive manufacturing industry was marked by intense and growing competition: “Existing and potential competitors may also have substantially greater financial, technical, marketing and sales, manufacturing, distribution, and other resources than us, including name recognition, as well as experience and expertise in intellectual property rights and operating within certain international markets or industry verticals, any of which may enable them to compete effectively against us.” The Markforged Holding Corporation (2021, p.41) also cautioned “that acquired technologies and intellectual property may be rendered obsolete or uneconomical by our own or our competitors' technological advances.”

The Markforged Holding Corporation (2021, p. 1) flagged the significance of intellectual property protection and enforcement: “We are, and have been in the recent past, subject to business and intellectual property litigation.” The Markforged Holding Corporation (2021, p. 1) noted: “If we are unable to adequately protect our proprietary technology or obtain and maintain patent protection for our technology and products or if the scope of the patent protection obtained is not sufficiently broad, our competitors could develop and commercialize technology and products similar or identical to ours, and our ability to successfully commercialize our technology and products may be impaired.”

The Markforged Holding Corporation (2021, p. 50) elaborated upon risks related to intellectual property litigation and liability.

The additive manufacturing industry has been, and may continue to be, litigious, particularly with respect to intellectual property claims. Moreover, our potential liabilities are subject to change over time due to new developments, changes in settlement strategy or the impact of evidentiary requirements. Regardless of the outcome, litigation has resulted in the past, and may result in the future, in significant legal expenses and require significant attention and resources of management. As a result, any present or future litigation that may be brought against us by any third party could result in reputational harm, losses, damages and expenses that may have a significant adverse effect on our financial condition.

The Markforged Holding Company mentions its intellectual property conflicts with Desktop Metal and Continuous Composites.

The Markforged Holding Corporation (2021, p. 56) discussed its approach to intellectual property management: “Our success is dependent, in part, upon protecting our proprietary information and technology.” The Markforged Holding Corporation (2021, p. 56) highlighted that the company relied upon a variety of forms of intellectual property: “Our intellectual property portfolio primarily consists of patents, patent applications, registered and unregistered trademarks, unregistered copyrights, domain names, know-how, and trade secrets.” The Markforged Holding Corporation (2021, p. 56) was conscious of the challenges in adequately protecting its intellectual property rights in its data and technology: “We may be unsuccessful in adequately protecting our intellectual property.”

The Markforged Holding Corporation (2021, p. 45) was also concerned about the international levels of intellectual property protection and enforcement, noting that there was “limited protection for the enforcement of contract and intellectual property rights in certain countries where we may sell our products or work with suppliers or other third parties.”

In addition to intellectual property litigation, the Markforged Holding Corporation (2021, p. 1) was also conscious of product liability claims: “We could be subject to personal injury, property damage, product liability, warranty and other claims involving allegedly defective products that we supply.” Moreover, the Markforged Holding Corporation (2021: 1) said: “We could face liability if our additive manufacturing solutions are used by our customers to print dangerous objects.”

Markforged Inc. (2022a) has made a number of disclosures of use of open source licensing. Markforged printers use “Flounder” firmware, which includes components of the Marlin open source project. A number of Markforged's furnaces, printers and desktop series include code from the Ubuntu open source project. Markforged's furnaces, printers and desktop series include code from the Debian GNU/Linux open source project.

## Patent litigation

There has been a growing scholarly literature in respect of patent law and 3D printing. Lemley (2015) has considered whether the patent system will be transformed by the super-abundance of things produced by 3D printing and other industry 4.0 technologies. Syzdek (2015) has charted a process of accommodation in patent jurisprudence of 3D printing. Daly (2016) has considered the socio-legal aspects of patent disputes over 3D printing. Van Overwalle and Leys (2017) have expressed confidence in the ability of the patent system to accommodate the disruptive influences of 3D printing. Mimler (2019) has considered whether United Kingdom patent law is ready for 3D printing.

Drawing comparisons with Napster, Desai and Magliocca (2014) wondered whether 3D printing and the digitization of things would result in mass patent infringement. Holbrook (2019) has explored remedies for digital patent infringement in the context of 3D printing. Nielsen and Nicol (2019) have considered Australian patent law and the emergence of 3D printing. Osborn (2019) has explored how United States patent law has been applied to the field of 3D printing. Griffin (2019) has looked at intellectual property, and the future of 3D printing, 4D printing, and augmented reality. Li (2014) has considered patent law and 3D bioprinting technologies. Ballardini et al. (2017) have considered the role of patent law in additive manufacturing in the EU. In this context, this paper makes an original contribution to this literature by focusing upon how patent law deals with a particular sub-field of 3D printing—namely, metal 3D printing.

### Complaint of patent infringement

In its 2021 Annual Report, Desktop Metal Inc. observed of its growing patent portfolio: “As of December 31, 2020, we own or co-own 34 issued United States patents, 25 issued foreign patents and have 143 pending or allowed patent applications” (Desktop Metal Inc, 2021, p. 16). The company indicated that its patents and its patent applications were directed to additive manufacturing and related technologies.

Desktop Metal filed a lawsuit against Markforged, Inc., alleging patent infringement *(Desktop Metal, Inc. v. Markforged, Inc. et al. D. Mass. Mar. 19, 2018. Docket 1:18-CV-10524)*. Ric Fulop commented: “Metal 3D printing is an exciting, quickly growing and rapidly evolving industry and, as a pioneer in the space, Desktop Metal welcomes healthy and vibrant competition” (Koslow, 2018). He observed: “When that competition infringes on our technology, however, we have a duty to respond” (Koslow, 2018). Fulop alleged: “We believe Markforged products clearly utilize technology patented by Desktop Metal and we will do what is necessary to protect our IP and our Company” (Koslow, 2018). James Coe, General Counsel of Desktop Metal, commented: “Desktop Metal has invested significant resources in developing innovative additive manufacturing technologies for metal 3D printing and our intellectual property portfolio reflects the hard work of our engineers and scientists” (Koslow, 2018). The lawyer maintained: “We owe it to our customers, employees and shareholders to protect the ground-breaking nature of our technology and preserve that investment so we can continue to promote innovation” (Koslow, 2018).

In its complaint, Desktop Metal discussed the development of its Studio System to manufacture 3D printed parts at scale:

In April 2017, Desktop Metal announced its Studio System, the first office-friendly metal 3D printing system for rapid prototyping, as well as its Production System to manufacture 3D printed parts at scale. The patented, proprietary Separable Supports used in Desktop Metal's 3D printing systems make it possible to remove support structures by hand. Desktop Metal's use of interface layers that allow for removable supports is unique to metal 3D printing (Desktop Metal., Inc., Complaint, 6).

In its complaint, Desktop Metal commented: “As Desktop Metal begins shipping its Studio System, Markforged is seeking to compete directly with Desktop Metal by offering its Metal X 3D print system” (Desktop Metal., Inc., Complaint, 9). The company observed: “Based on at least Markforged's recent disclosures that its Metal X 3D print system uses a ceramic release layer that turns to powder during sintering, Markforged seeks to compete using Desktop Metal's patented technology protected by the Patents-in-Suit” (Desktop Metal., Inc., Complaint, 9).

Desktop Metal Inc. highlighted its patent for “fabricating an interface layer for removable support,” U.S. Patent No. 9,833,839 B2 (Gibson et al., 2017). Desktop Metal Inc. alleged that “Markforged has infringed and continues to infringe, directly and indirectly by way of inducement and/or contributory infringement, one or more claims of the '839 patent” (Desktop Metal., Inc., Complaint, 10). Desktop Metal Inc. alleged: “Markforged has infringed at least claim 1 of the '839 patent through use of its Markforged Metal X 3D print system to practice the patented method for fabricating, from a first material, a support structure for an object; fabricating an interface layer adjacent to the support structure; and fabricating a surface of the object from a second material, the surface of the object adjacent to the interface layer and the second material including a powdered material for forming a final part and a binder system including one or more binders, wherein the one or more binders retain a net shape of the object during processing of the object into the final part, wherein processing of the object into the final part includes debinding the net shape to remove at least a portion of one or more binders and sintering the net shape to join and densify the powdered material, and wherein the interface layer resists bonding of the support resists bonding of the support structure to the object during sintering” (Desktop Metal., Inc., Complaint, 10). “Markforged's infringement has caused and is continuing to cause damage and irreparable injury to Desktop Metal” (Desktop Metal., Inc., Complaint, 15). Desktop Metal Inc. sought injunctive relief and damages for the alleged patent infringement. Desktop Metal Inc. also sought enhanced damages on the basis that Markforged's conduct amounted to willful patent infringement.

Desktop Metal Inc. also highlighted its patent for “Fabricating Multi-Part Assemblies,” U.S. Patent No. 9,815,118 B1 (Schmitt et al., 2017). Desktop Metal Inc. argued: “Markforged has infringed and continues to infringe, directly and indirectly by way of inducement and/or contributory infringement, one or more claims of the '118 patent” (Desktop Metal., Inc., Complaint, 16). Desktop Metal Inc. contended: “Markforged has infringed at least claim 1 of the '118 patent through use of its Markforged Metal X 3D print system to practice the patented method for fabricating a first object from a first material, wherein the first material includes a powdered material and a binder system, the binder system including one or more binders that resist deformation of a net shape of the first object during processing of the first object into a final part; applying an interface layer adjacent to a first surface of the first object; and fabricating a second surface of a second object from a second material at a location adjacent to the interface layer and opposing the first surface of the first object, wherein the second object is structurally independent from and mechanically related to the first object, wherein the interface layer resists bonding of the first surface to the second surface during sintering, and wherein the interface layer reduces to a powder during sintering of the first material” (Desktop Metal., Inc., Complaint, 16–17). Desktop Metal Inc. sought injunctive relief and damages in respect of the alleged patent infringement. Desktop Metal Inc. also asked for a finding of willful infringement, and sought enhanced damages.

In its prayer for relief, Desktop Metal Inc. sought “A declaration in favor of Desktop Metal and against Markforged on each count of this Complaint, and a final judgment incorporating the same (Desktop Metal., Inc., Complaint, 31). Desktop Metal Inc. asked the court for ‘a preliminary and permanent injunction, enjoining Markforged and its officers, agents, servants, employees, representatives, successors, and assigns, and all others acting in concert or participation with them from continued infringement of the ’839 patent and '118 patent, under 35 U.S.C. § 283” the '839 patent and '118 patent, under 35 U.S.C. § 283” (Desktop Metal., Inc., Complaint, 31–32). Desktop Metal Inc. soug “An award of damages adequate to compensate Desktop Metal for Markforged's infringement the '839 patent and '118 patent, together with pre- and post-judgment interest and costs pursuant to 35 U.S.C. § 284” (Desktop Metal., Inc., Complaint, 32). Desktop Metal Inc. requested “An order finding that Markforged's infringement is willful and enhancing damages pursuant to 35 U.S.C. § 284” (Desktop Metal., Inc., Complaint, 32). Desktop Metal Inc. also sought “an order finding that this is an exceptional case under 35 U.S.C. § 285” (Desktop Metal., Inc., Complaint, 32). Desktop Metal Inc. asked for “an accounting of all infringing sales and other infringing acts by Markforged, and an order compelling an accounting for infringing acts not presented at trial and an award by the Court of additional damages.

### Response by markforged

After initially declining to issue a media statement, Greg Mark of Markforged Inc. issued a statement about the litigation:

I founded Markforged in my kitchen 6 years ago. I dreamt of giving every engineer the ability to 3D print real, functional, mechanical parts. We invented something that had never existed before—a continuous carbon fiber 3D printer. Our Metal X product is an extension of that platform. We've come a long way. We now have the most advanced technology platform in 3D printing, and I'm incredibly proud of what our team of engineers have accomplished (Koslow, 2018).

Mark noted that “a competitor filed a lawsuit against us, including various far-fetched allegations” (Koslow, 2018). Mark observed: “Markforged categorically denies these allegations and we will be formally responding shortly in our own court filing” (Koslow, 2018). He maintained: “Markforged is a thriving business with a dedicated team of passionate people, and we're going to continue to execute and deliver amazing products to our customers” (Koslow, 2018).

In their answer to the complaint of Desktop Metal Inc., Markforged Inc. was indignant at the allegations of patent infringement, trade secrets violations, and other forms of intellectual property infringement, denying that it had committed such offenses. It also noted that the allegations of breaches of contract law and consumer law were directed toward a third party. Markforged Inc. initially listed a catalog of twenty-five defenses to the complaint by Desktop Metal.

In its first defense, Markforged Inc. maintained: “Desktop Metal's claims are barred in whole or in part because Markforged has not directly infringed, induced infringement, or contributed to infringement, and does not directly infringe, induce infringement, or contribute to infringement, of any valid and enforceable claim of the Asserted Patents, either literally or under the doctrine of equivalents, and has not otherwise committed any acts in violation of 35 U.S.C. § 271” *(Markforged Inc's Answer)*.

In its second defense, Markforged Inc. argued: “Desktop Metal's claims are barred in whole or in part because one or more claims of the Asserted Patents are invalid for failure to comply with one or more of the requirements of the Patent Laws of the United States, 35 U.S.C. §§ 100, et seq., including, but not limited to, §§ 101, 102, 103, and/or 112” *(Markforged Inc's Answer)*. In its view, “The invalidity of certain asserted claims is demonstrated, for example, by at least prior art references US 2015/0197862 A1 and US 2015/0306664 A1” *(Markforged Inc's Answer)*.

In its third defense, Markforged Inc. maintained that Desktop Metal's claims were barred in whole or in part by reason of estoppel.

Fourth, Markforged Inc. argued that “Desktop Metal's claims are barred in whole or in part because Markforged has a license to the Asserted Patents” *(Markforged Inc's Answer)*. The company maintained: “Under the Terms of Service and Software End User License Agreement, to which Desktop Metal and its employee agreed at the time of the sale, Desktop Metal has granted to Markforged a fully paid-up, royalty-free, worldwide, non-exclusive, irrevocable, transferable license in, under, and to the Asserted Patents” *(Markforged Inc's Answer)*.

Fifth, Markforged Inc. argued that “The Asserted Patents are unenforceable due to inequitable conduct by the inventors, prosecuting attorneys, or both, in failing to discharge their duty of candor to the United States Patent and Trademark Office (‘USPTO’)” *(Markforged Inc's Answer)*. The company observed: “On information and belief, Desktop Metal's patent prosecution counsel, the inventors of the Asserted Patents, or both, knowingly omitted or made affirmative misrepresentations of material information to the USPTO with a specific intent to deceive the USPTO” *(Markforged Inc's Answer)*.

Sixth, Markforged Inc. alleged “Desktop Metal is not entitled to injunctive relief or enhanced damages because it failed to plead the required elements for such relief, and because Desktop Metal has an adequate remedy at law for any alleged injury” *(Markforged Inc's Answer)*.

Seventh, Markforged Inc. maintained that “Desktop Metal's claims are barred in whole or in part by 35 U.S.C. §§ 286, 287 or 288” *(Markforged Inc's Answer)*.

Eighth, Markforged Inc. contended that “One or more of Desktop Metal's claims are barred by the doctrine of unclean hands” *(Markforged Inc's Answer)*. In particular, it argued: “As just one example, Desktop Metal acquired the information it used to file and obtain the Asserted Patents as the result of a series of unlawful and deceptive acts” *(Markforged Inc's Answer)*.

Ninth, Markforged Inc. denied that there had been any damage suffered by Desktop Metal. Tenth, Markforged Inc. insisted that Desktop Metal's claims were barred by the doctrines of laches and estoppel. Eleventh, Markforged Inc. maintained that Desktop Metal's “claims are frivolous, brought in bad faith and/or are brought for an improper purpose and/or were brought without reasonable inquiry” *(Markforged Inc's Answer)*. Twelfth, Markforged Inc. argued: “Desktop Metal's claims are barred in whole or in part because it is unable to establish that Markforged caused any of the harm for which it is seeking redress” *(Markforged Inc's Answer)*. The thirteenth defense was that Desktop Metal had waived any rights or claims it may have against Markforged. The fourteenth defense was that Desktop Metal had failed to mitigate any damages claims it may have against Markforged. Fifteenth defense was that Desktop Metal's complaint fails to state a claim upon which relief can be granted. The sixteenth defense was that the plaintiff's claims are barred under the doctrine of *in pari delicto*.

### Patent infringement trial

The presiding judge was Justice William G. Young—a senior judge in the United States District Court for the District of Massachusetts. Having studied by Harvard Law School, Young received his commission in 1985; served as a chief judge between 1999 and 2005; and assumed his senior status in 2021.

The case was brought in front of a 12-person federal jury in Boston on Monday 9th July 2018 (Jackson, 2018b). The parties engaged in extensive argument about patent validity and patent infringement. The parties also drafted their preferred version of jury instructions. After 3 weeks on trial, the jury reached the verdict around 10 a.m. on Friday 27th July 2018 (Jackson, 2018b). On the 27th July 2018, the Jury handed down its verdict in the dispute between Desktop Metal Inc. and Markforged Inc. on patent validity and patent infringement [*Desktop Metal, Inc. v. Markforged, Inc. 2018 4007724 (D. Mass.) (Verdict, Agreement and Settlement)*].

### Jury verdict

Question 1: “118 Patent. With respect to the claims in the '*118 Patent* (answer ‘yes’ or ‘no’ in each box”):

**Table:** 

	Validity		Infringed?		
	**Anticipated?**	**Obvious?**	**Indefinite?**	**Direct?**	**Indirect?**
Claim 1	No	No	No	No	No
Claim 2	No	No	No	No	No
Claim 3	No	No	No	No	No
Claim 4	No	No	No	No	No
Claim 10	No	No	No	No	No
Claim 11	No	No	No	No	No
Claim 12	No	No	No	No	No
Claim 13	No	No	No	No	No
Claim 14	No	No	No	No	No
Claim 17	No	No	No	No	No
Claim 24	No	No	No	No	No

Question 2: “839 Patent. With respect to the claims in the '*839 Patent* (answer ‘yes’ or ‘no’ in each box”):

**Table:** 

	Validity		Infringed?		
	**Anticipated?**	**Obvious?**	**Indefinite?**	**Direct?**	**Indirect?**
Claim 1	No	No	No	No	No
Claim 2	No	No	No	No	No
Claim 3	No	No	No	No	No
Claim 4	No	No	No	No	No
Claim 10	No	No	No	No	No
Claim 16	No	No	No	No	No
Claim 17	No	No	No	No	No
Claim 18	No	No	No	No	No
Claim 20	No	No	No	No	No
Claim 21	No	No	No	No	No
Claim 23	No	No	No	No	No

Question 3: Willful Infringement. If you find that Markforged infringed one or more of the claims in either patent, was the infringement willful? Answer “yes” or “no.”

Answer: No

Question 4: If you find that Markforged infringed one or more of the valid claims in either patent, what amount of money damages (in U.S. dollars) for lost profits do you award to Desktop Metal?

Damages: $ 0

<<signature>>
Forelady
Date: 7/27/18

Greg Mark, CEO of Markforged Inc., commented on the outcome: “Markforged printers have changed the way businesses produce strong parts while dramatically impacting the delivery times, cost, and supply chain logistics” (Koslow, 2018). He observed: “We feel gratified that the jury found we do not infringe, and confirmed that the Metal X, our latest extension of the Markforged printing platform, is based on our own proprietary Markforged technology” (Koslow, 2018).

Desktop Metal commented on the outcome of the jury trial in respect of patent validity and infringement:

Desktop Metal is pleased that the jury agreed with the validity of all claims in both of Desktop Metal's patents asserted against Markforged. Desktop Metal has additional claims pending alleging trade secret misappropriation by Markforged. The Federal District Court has bifurcated those counts and will try them at a later date. At Desktop Metal, we remain committed to building on our leadership in the metal 3D printing sector and continuing to provide innovative products and solutions to our hundreds of customers across industries (Koslow, 2018).

Desktop Metal observed that they were seeking further legal advice about the finding of no patent infringement: “We are currently reviewing legal options concerning the infringement issue” (Koslow, 2018). Raymond and Wolfe (2018) reported for *Reuters*: “A federal jury on Friday found metal 3D printing systems maker Markforged Inc did not infringe two patents held by rival Desktop Metal Inc, delivering a verdict that could determine leadership in the nascent market for the companies' products.”

## Trade secrets litigation

In addition to a patent dispute between Desktop Metal and Markforged, there was also a contentious dispute over trade secrets and confidential information, and other related matters associated with unfair competition and contract law.

As Lemley (2008) has noted, the field of trade secrets is puzzling, defying easy categorization in terms of its disciplinary identity (with various influences, ranging from contract law, property law, equity law, employment law, and human rights). Nonetheless, it is productive and helpful to consider trade secrets as a species of intellectual property, sitting alongside the various other forms of intellectual property. There has been a dramatic expansion of growth of trade secrets law in the United States of late (Rowe and Sandeen, 2021).

There has been an increasing interest in the use of trade secrets and confidential information in the field of 3D printing and additive manufacturing (Mendis et al., 2019, p. 376–379). Vogel (2016, p. 896) commented that trade secrets would be a useful alternative to patent protection: “In addition to easier burdens of proof and no filing requirement, trade secret provides ample protection against the potential exploitation of the industry's valuable proprietary information.” He emphasized that trade secrets protection was particularly important in the “quickly evolving, growing, and consolidating field of additive manufacturing” (Vogel, 2016, p. 898). Vogel (2016, p. 898) also noted the limitations of the regime: “While trade secret law can protect against misappropriation of proprietary processes and methods, this protection is less robust than that available under patent law.” He also acknowledged that “detecting and proving misappropriation in the complex and rapidly changing additive manufacturing arena can be challenging (Vogel, 2016, p. 898).”

There have been some early skirmishes over trade secrets and confidential information in the field of 3D printing. In 2016, the 3D printing company Magic Leap sued two of its former employees for trade secret misappropriation in the United States (Molinski and Heath, 2016). In 2017, the judge ruled that Magic Leap failed to disclose the trade secrets with sufficient particularity *(**Magic Leap Inc. v Bradski et al. Case Number 5:16-cvb-02852*., *2017**)*. The dispute was settled between the parties in August 2017 (Pounds, 2017).

In his book on additive manufacturing of metals, Milewski (2017, p. 283) has commented: “Trade secret law is evolving in an attempt to keep up with information, privacy, cyber security, hacking and a highly mobile, global workplace.” He noted: “The U.S. Government is enacting laws such as the *Defend Trade Secrets Act* of 2016 to mitigate the problem” (2017, p. 283). Milewski observed: “Industrial espionage will increase as will the efforts and methods used to counter these threats” (2017, p. 283). Trade secrets may well have a heightened application in the field of metal 3D printing.

There has been some disquiet about the rapid expansion of trade secrets law at a policy level. Lobel (2013) has worried that the over-protection of trade secrets has had an adverse impact on innovation, competition, and the mobility of labor. Menell (2017) has argued that there is a need to develop clear defenses, limitations, and exceptions in respect of trade secrets law. Hrdy and Lemley (2021) have argued that there should be a doctrine of trade secrets abandonment to better protect and preserve the public domain.

It is also worth noting that bilateral and regional trade agreements—such as the *Trans-Pacific Partnership* 2015—have been seeking to raise the standards of protection for trade secrets internationally (Rimmer, 2020b, p. 380–411).

### The trade secrets of desktop metal Inc.

In its 2021 annual report, Desktop Metal Inc. details the importance of trade secrets and confidential information to its business:

Our trade secrets, know-how and other unregistered proprietary rights are a key aspect of our intellectual property portfolio. While we take reasonable steps to protect our trade secrets and confidential information and enter into confidentiality and invention assignment agreements intended to protect such rights, such agreements can be difficult and costly to enforce or may not provide adequate remedies if violated, and we may not have entered into such agreements with all relevant parties. Such agreements may be breached, and trade secrets or confidential information may be willfully or unintentionally disclosed, including by employees who may leave our company and join our competitors, or our competitors or other parties may learn of the information in some other way (Desktop Metal Inc, 2021, p. 36).

Desktop Metal Inc. observed: “The disclosure to, or independent development by, a competitor of any of our trade secrets, know-how or other technology not protected by a patent or other intellectual property system could materially reduce or eliminate any competitive advantage that we may have over such competitor” (Desktop Metal Inc, 2021, p. 36). Desktop Metal Inc. was particularly concerned about its consumable products: “This concern could manifest itself in particular with respect to our proprietary consumables that are used with our systems” (Desktop Metal Inc, 2021, p. 36). Desktop Metal Inc. observed that its patent protection did have limits and boundaries: “Portions of our proprietary consumables may not be afforded patent protection” (Desktop Metal Inc, 2021, p. 36).

Desktop Metal Inc. cautions: “Chemical companies or other producers of raw materials used in our consumables may be able to develop consumables that are compatible to a large extent with our products, whether independently or in contravention of our trade secret rights and related proprietary and contractual rights” (Desktop Metal Inc, 2021, p. 36). Desktop Metal Inc. fears: “If such consumables are made available to owners of our systems, and are purchased in place of our proprietary consumables, our revenues and profitability would be reduced, and we could be forced to reduce prices for our proprietary consumables” (Desktop Metal Inc, 2021, p. 36).

### Desktop metal complaint

In its complaint, Desktop Metal Inc. alleged that an intern Mr Parangi had a familial relationship to a Markforged employee, and that Markforged had engaged in trade secret misappropriation, unfair and deceptive business practices, and breach of contract (Desktop Metal., Inc., Complaint, 21–24). Desktop Metal Inc. alleged: “Mr. Parangi's relation to Abraham Parangi caused Desktop Metal to become suspicious that he may have been involved in sharing Desktop Metal's Proprietary Information with Markforged” (Desktop Metal., Inc., Complaint, 23). Desktop Metal Inc. alleged: “Based on this investigation, Desktop Metal learned that on October 20, 2016, Mr. Parangi had downloaded documents unrelated to his work on the print farm, including documents containing Proprietary Information such as a document titled ‘Engineer Status and Goals-160912’ which at the time, provided a snapshot of the status of some of the research projects within the Desktop Metal, as well as the next steps for key personnel” (Desktop Metal., Inc., Complaint, 24). Desktop Metal Inc. argued that “Mr. Parangi misappropriated Desktop Metal's Proprietary Information and passed them along to his brother and/or others at Markforged” and “Markforged, with full knowledge that a Desktop Metal employee had misappropriated the Proprietary Information, then used that information in developing a metal 3D printing process that mimics Desktop Metal's approach” (Desktop Metal., Inc., Complaint, 24).

Desktop Metal Inc. alleged that Mr. Parangi and Markforged Inc. had violated the *Defend Trade Secrets Act* of 2016 (US). The company noted: “Desktop Metal has expended significant resources to develop its trade secrets to offer a unique and revolutionary metal 3D printing process” (Desktop Metal., Inc., Complaint, 25). The company stressed: “Desktop Metal's trade secrets derive independent economic value, actual or potential, from not being generally known to, and not being readily ascertainable through proper means by, another person who can obtain economic value from the disclosure or use of the information” (Desktop Metal., Inc., Complaint, 25). the information” (Desktop Metal., Inc., Complaint, 25). The company stressed: “These trade secrets are highly valuable to Desktop Metal and to any other person or entity that wants to enter the field of 3D metal printing” (Desktop Metal., Inc., Complaint, 25). Desktop Metal Inc. alleged that “Mr. Parangi knew, or had reason to know, that he had acquired trade secrets from Desktop Metal through improper means, and disclosed Desktop Metal's trade secrets, in direct violation of his express obligations to Desktop Metal, to his brother and/or others at Markforged” (Desktop Metal., Inc., Complaint, 25). Desktop Metal Inc. alleged that “Markforged knew, or had reason to know, that it acquired trade secrets from Desktop Metal through improper means and used Desktop Metal's trade secrets without Desktop Metal's consent, knowing or having reason to know that the trade secrets were acquired by improper means” (Desktop Metal., Inc., Complaint, 25). Desktop Metal Inc. sought various remedies—including civil seizure of property, injunctive relief, monetary damages for its actual losses, and monetary damages for unjust enrichment. Desktop Metal Inc. also maintained that the misappropriation was willful Rehearsing similar allegations, Desktop Metal Inc. also accused Mr. Parangi and Markforged of trade secret misappropriation: “As a direct and proximate result of Mr. Parangi's and Markforged's misappropriation of trade secrets, Desktop Metal has suffered and will continue to suffer irreparable harm and other damages, including, but not limited to, loss of value of its trade secrets” (Desktop Metal., Inc., Complaint, 26).

Desktop Metal Inc. also accused Mr. Parangi and Markforged of unfair and deceptive trade practices. Highlighting the non-disclosure agreement that its intern signed, Desktop Metal Inc. argued: “On information and belief, in direct violation of his contractual obligations to Desktop Metal, Mr. Parangi disclosed Desktop Metal's Proprietary Information to his brother and/or others at Markforged, assisting Markforged to develop a directly competing product in the 3D metal printing field” (Desktop Metal., Inc., Complaint, 28). Desktop Metal Inc. contended: “On information and belief, Markforged knowingly received the benefits from the disclosure of Desktop Metal's Proprietary Information and used it to develop a directly competing product in the 3D metal printing field” field” (Desktop Metal., Inc., Complaint, 28). Desktop Metal Inc. argued: “The aforementioned acts and practices of Mr. Parangi and Markforged constitute unfair methods of competition or unfair or deceptive acts or practices that occurred primarily and substantially within Massachusetts within the meaning of M.G.L. c. (Desktop Metal., Inc., Complaint, 28).

Furthermore, Desktop Metal Inc. alleged that there had been a breach of contract of the non-disclosure agreement: “Mr. Parangi breached his contractual obligations to Desktop Metal under the NDA by downloading Desktop Metal's Proprietary Information and removing the downloaded materials from Desktop Metal's premises” (Desktop Metal., Inc., Complaint, 29). Desktop Metal Inc. also argued that there had been a breach of a Non-Competition and Non-Solicitation Agreement: “Mr. Parangi breached his contractual obligations to Desktop Metal under the Non-Competition and Non-Solicitation Agreement by passing along Desktop Metal's Proprietary Information to his brother and/or others at Markforged, assisting Markforged to develop a directly competing product in the 3D in the 3D metal printing field” (Desktop Metal., Inc., Complaint, 30). It should be noted that there has been much academic debate about the use of non-compete clauses in relation to intellectual property (Lobel, 2013; Bessen, 2015; Sandeen and Rowe, 2017; Lemley and Lobel, 2021). The Biden Administration has issued an executive order, calling on a curtailment of non-compete clauses (White House, 2021).

Finally, Desktop Metal Inc. alleged that there had been a breach of the covenant of good faith and fair dealing: “Mr. Parangi has, through improper means and in bad faith, used and/or disclosed Desktop Metal's Proprietary Information in an effort to benefit his brother and Markforged, in direct violation of his express obligations” (Desktop Metal., Inc., Complaint, 31).

Desktop Metal Inc. protested: “Mr. Parangi did not reveal that his brother was a senior engineer at Markforged until directly asked whether this was true” (Desktop Metal., Inc., Complaint, 31). Desktop Metal Inc. argued: “By acting through improper means and in bad faith, Mr. Parangi has deprived Desktop Metal of the benefits owed to it under the contracts” (Desktop Metal., Inc., Complaint, 31).

### Response of markforged inc.

In its answer, Markforged Inc. made a number of responses to the trade secrets claims (Markforged, Answer).

Seventeenth, “Plaintiff's claims alleging misappropriation of trade secrets are barred, in whole or in part, because the information allegedly misappropriated was readily ascertainable by proper means” (Markforged, Answer). Eighteenth, “Plaintiff's claims alleging misappropriation of trade secrets are barred, in whole or in part, because Plaintiff did not take proper efforts to keep the information secret” (Markforged, Answer) (The nineteenth defense seemed to be missing from this initial document).

Twentieth, “Plaintiff's claims against Markforged alleging misappropriation of trade secrets and unfair competition are barred, in whole or in part, because Markforged did not obtain any purported trade secrets or confidential information by improper means” (Markforged, Answer). Twenty-first defense was that the “Plaintiff's claims against Markforged alleging misappropriation of trade secrets and unfair competition are barred, in whole or in part, because Markforged has not used and is not using any of Plaintiffs' alleged trade secrets or confidential information” (Markforged, Answer).

The 22nd defense was that the “Plaintiff's claims alleging misappropriation of trade secrets are barred, in whole or in part, by Markforged's independent development” (Markforged, Answer). The 23rd defense was that the “Plaintiff's claims alleging misappropriation of trade secrets are barred, in whole or in part, because the alleged trade secrets or confidential information lack independent economic value” (Markforged, Answer). The 24th defense was that the “Plaintiff's claims alleging misappropriation of trade secrets are barred, in whole or in part, because Plaintiff's alleged trade secrets have not been in continuous use” (Markforged, Answer). The 25th defense was that the “Plaintiffs' claims alleging misappropriation of trade secrets and unfair competition are barred, in whole or in part, to the extent they are preempted by federal law” (Markforged, Answer).

### Counterclaims of markforged

The Markforged Holding Corporation (2021, p. 57) has discussed the importance of trade secrets in its corporate filings: “Our trade secrets, know-how and other unregistered proprietary rights are a key aspect of our intellectual property portfolio.” The Markforged Holding Corporation (2021, p. 57) observed: “While we take reasonable steps to protect our trade secrets and confidential information and enter into confidentiality and invention assignment agreements intended to protect such rights, such agreements can be difficult and costly to enforce or may not provide adequate remedies if violated, and we may not have entered into such agreements with all relevant parties.” The Markforged Holding Corporation (2021, p. 57) was conscious of the dangers of the breach of confidential information: “Such agreements may be breached and trade secrets or confidential information may be willfully or unintentionally disclosed, including by employees who may leave our company and join our competitors, or our competitors or other parties may learn of the information in some other way.” The Markforged Holding Corporation (2021, p. 57) commented: “Additionally, certain unauthorized use of our intellectual property may go undetected, or we may face legal or practical barriers to enforcing our legal rights even where unauthorized use is detected.”

The Markforged Holding Corporation (2021, p. 57) cautioned about a particular scenario: “Chemical companies or other producers of raw materials used in our materials may be able to develop materials that are compatible to a large extent with our products, whether independently or in contravention of our trade secret rights and related proprietary and contractual rights.” The Markforged Holding Corporation (2021, p. 57) observed: “If such materials are made available to owners of our systems, and are purchased in place of our proprietary materials, our revenues and profitability would be reduced, and we could be forced to reduce prices for our proprietary materials.”

As well as making a defense against Desktop Metal, Markforged Inc. also made a number of counterclaims against its rival (Markforged Inc., Counterclaims, 2018). Markforged Inc. argued: “Desktop Metal has had the temerity to sue Markforged even though it is the product of the unscrupulous and deceptive conduct of Ric Fulop and his long-time friend and business partner Jonah Myerberg” (Markforged Inc., Counterclaims, 2018, 23). The company noted: “Fulop joined Markforged at virtually the beginning, providing key financing from his firm and becoming a Director in June 2013” (Markforged Inc., Counterclaims, 2018, 23). Markforged Inc. maintained: “Once ensconced at Desktop Metal, Fulop continued to engage in unfair acts and conduct, taking key employees and prospects from Markforged, falsely disparaging Markforged in the marketplace as a manufacturer of cheap plastic 3D printers, and even causing Third-Party Defendant and employee of Desktop Metal, Amy Buntel, to engage in the ruse of purchasing a Markforged 3D printer and having it shipped to her home so that Fulop, Myerberg, and others at Desktop Metal could disassemble, analyze and use it in order to prepare their own patent applications based on Markforged's product and technology” (Markforged Inc., Counterclaims, 2018, 24).

In its Counterclaims, Markforged Inc. claimed that Fulop, Myerberg, and Desktop Metal were in Violation of the Defend Trade Secrets Act. The company commented:

Markforged has expended significant resources to develop its trade secrets and other confidential and proprietary information, to offer a unique and revolutionary way to 3D print high-strength parts on a desktop. Markforged's trade secrets and confidential and proprietary information, derive independent economic value, actual or potential, from not being generally known to, and not being readily ascertainable through proper means by, another person who can obtain economic value from the disclosure or use of the information. These trade secrets and confidential, proprietary information are highly valuable to Markforged and to any other person or entity that wants to enter the field of 3D printing high-strength parts in a desktop environment (Markforged Inc., Counterclaims, 2018, 42).

The company observed: “Ric Fulop knew, or had reason to know, that he had acquired trade secrets from Markforged through improper means, and disclosed Markforged's trade secrets to Desktop Metal and others, in direct violation of his fiduciary obligations to Markforged” (Markforged Inc., Counterclaims, 2018, 42). Markforged Inc. also alleged that Jonah Myerberg and Boston Impact and Desktop Metal Inc. misappropriated its trade secrets.

Markforged Inc. also alleged that Fulop, Myerberg, and Desktop Metal had engaged in misappropriation of trade secrets and confidential information. The company alleged: “Ric Fulop stole or unlawfully took, carried away, concealed, and/or copied trade secrets and other confidential proprietary information from Markforged and disclosed Markforged's trade secrets and other confidential proprietary information to Desktop Metal, in direct violation of his fiduciary obligations to Markforged” (Markforged Inc., Counterclaims, 2018, 48). There were similar allegations about Myerberg, Boston Impact, and Desktop Metal in respect of trade secrets and confidential information.

Markforged Inc. accused Fulop of a breach of fiduciary duty: “Fulop's unlawful conduct has injured Markforged's 3D printing business, and will continue to harm Markforged's business until Fulop's efforts are curtailed” (Markforged Inc., Counterclaims, 2018, 56). Markforged Inc. alleged that Desktop Metal Inc. had aided and abetted a breach of fiduciary duty.

Markforged Inc. lodged accusations of unfair business methods by Fulop, Myerberg. Buntel and Desktop Metal (Markforged Inc., Counterclaims, 2018, 58). The company alleged: “Ric Fulop engaged in a course of conduct designed to unfairly harm Markforged, to Desktop Metal's advantage, through his business transactions with Markforged” (Markforged Inc., Counterclaims, 2018, 59).

Markforged Inc. accused a number of parties of breach of contract (Boston Impact, Buntel, Desktop Metal) and aiding and abetting breach of contract (Fulop, Desktop Metal). There was also an accusation of a breach of covenant of good faith and fair dealing (Boston Impact). Markforged Inc. also alleged tortious interference with advantageous contractual/relations (Fulop, Desktop Metal), and prospective contractual relations (Fulop, Desktop Metal).

Markforged Inc. claimed that there had been a civil conspiracy: “Desktop Metal, Ric Fulop, and Jonah Myerberg engaged in overt actions to further this conspiracy, including but not limited to Ric Fulop using confidential information about Markforged's key potential hires to poach and recruit those individuals for Desktop Metal, and Jonah Myerberg, by and through his company Boston Impact, accepting a position as a beta tester at Markforged in order to gain access to confidential, proprietary and trade secret information about Markforged's 3D printing products, and to use that information to advance Desktop Metal's 3D printing products” (Markforged Inc., Counterclaims, 2018, 77).

Markforged Inc. also accused a number of parties of unjust enrichment (Fulop, Myerberg, Boston Impact, Desktop Metal). The company alleged: “But for Ric Fulop's unjust and inequitable conduct, Markforged would have obtained additional investor funding, maintained additional 3D printer customers, maintained its trade secret and confidential proprietary information, and maintained its position as the only 3D printing company offering a printer that can produce high-strength parts on a desktop, and at an accessible price point” (Markforged Inc., Counterclaims, 2021., 78).

## Trial, settlement, and arbitration

The dispute between Desktop Metal Inc. and Markforged Inc. was briefly aired with a trial—but that was halted, with a settlement between the parties. There was a further dispute between the parties as to whether there had been a breach of a settlement—but an arbitration ruling found that there had been no breach of the settlement by the parties.

### Trial

There was a 2-week trial scheduled for the trade secrets litigation and associated matters in September 2018. O'Brien (2018a,b,c,d) provided excellent coverage of the trial in a series of insightful pieces for the *Boston Business Journal*.

O'Brien (2018a) previewed the dispute: “A trial is set to begin next week in a trade secrets lawsuit in which two of Massachusetts' top industrial 3D-printing startups accuse each other of lying, stealing, spreading rumors and planting spies.” O'Brien (2018a) commented that the “The trial… will provide a rare look behind the scenes at two competitors fighting to capture a burgeoning market that could be worth billions of dollars per year.” O'Brien (2018a) also noted that the dispute and the trial “will also map some of the key relationships within Boston's close-knit community of venture capital investors, tech executives and university researchers.”

Providing an eyewitness account of the dispute, O'Brien (2018b) observed: “The opening statements at the Seaport's Moakley courthouse outlined the key questions in the trade secrets battle between Markforged and Desktop Metal, which are both promising to revolutionize the manufacturing process by making it faster and cheaper to create complicated parts out of metal or other industrial-strength materials.” The opening statements by the parties highlighted the intense competition between the two companies. O'Brien (2018b) highlighted what was at stake in the dispute: “The market could be worth billions of dollars per year, and the opportunity has spurred the companies to raise more than $325 million in combined investor funding.”

Going beyond the extracts reported in the media, the trial transcripts provide a good sense of the narratives of the competing parties *(**Desktop Metal Inc. v Markforged Inc., Case 1:18-cv-10524-WGY, Document 560, Trade Secrets Trial, Transcripts**)*.

Acting for Markforged Inc., Harvey Wolkoff made this opening statement to the jury:

You're going to hear that this is a case about disloyalty and betrayal. Ric Fulop was on the board of directors of Markforged, which is a 3D printing company that was started by Greg Mark. As a board member, Ric Fulop owed Markforged under the law fiduciary duties of loyalty and of honesty. But instead what you'll hear is that Ric Fulop started a competing 3D printing company called Desktop Metal while he was sitting on the Markforged board. And more than that you're going to hear that he hid what he was doing, hid it because he knew it was wrong *(**Desktop Metal Inc. v Markforged Inc., Case 1:18-cv-10524-WGY, Document 560, Trade Secrets Trial, Transcripts*, 27).

Wolkoff concluded his opening address: “I'm going to ask you to award Markforged its damages from this betrayal, from this breach of fiduciary duties, from this breach of the obligation to have your utmost loyalty to Markforged.” (*Desktop Metal Inc. v Markforged Inc., Case 1:18-cv-10524-WGY, Document 560, Trade Secrets Trial, Transcripts*, 38–39).

Representing Mr. Parangi, Mr. Ward argued that his client had been mistakenly drawn into the dispute between the two metal 3D printing companies:

The evidence will show that when Markforged did build its first 3D metal printer, the Metal X, it didn't even use any of these so-called trade secrets that Desktop Metal s ays came from Matiu. And anyway you'll hear at trial of the supposed trade secrets. There's a lot of publicly-available information that's well-known to people in the industry. They weren't even trade secrets at all *(**Desktop Metal Inc. v Markforged Inc., Case 1:18-cv-10524-WGY, Document 560, Trade Secrets Trial, Transcripts*, 43–44).

Mr. Ward implored the jury: “Ladies and gentlemen, as the evidence will show this case against Matiu Parangi is entirely speculative” (Desktop Metal Inc. v Markforged Inc., Case 1:18-cv-10524-WGY, Document 560, Trade Secrets Trial, Transcripts, 44). Mr Ward argued: “Just because Matiu's brother happened to work for Markforged, Desktop Metal has accused him of stealing their trade secrets” (*Desktop Metal Inc. v Markforged Inc., Case 1:18-cv-10524-WGY, Document 560, Trade Secrets Trial, Transcripts*, 44). Mr Ward wrapped up his opening statement, claiming: “This is really a case of one company against another company, and I submit to you Matiu Parangi shouldn't be dragged into this at all.” (*Desktop Metal Inc. v Markforged Inc., Case 1:18-cv-10524-WGY, Document 560, Trade Secrets Trial, Transcripts*, 44).

Appearing for Desktop Metal Inc., Ms Lynne Hermle maintained that Ric Fulop did not breach of any fiduciary duty to Markforged Inc.

Ric Fulop did not breach any fiduciary duty to Markforged because there was nothing secret and certainly nothing inappropriate about his behavior. In fact he offered Markforged the opportunity for the metal concept in printing that he had first. Time after time after time he urged Greg Mark to move Markforged toward metal printing, which he believed would be a great opportunity for this company in which he had invested. The e-mails that you saw, which were highlighted only in part, you'll be able to read the entire e-mails and to see that over and over and over again he said to Greg Mark that Markforged should go into the metal printing business. You won't have to take my word for it, when you see the e-mails and are able to read all of them, you'll see that over and over again he urges Markforged to have employees working on metal, and you'll even see that Greg Mark criticizes him and makes fun of him for that advice (*Desktop Metal Inc. v Markforged Inc., Case 1:18-cv-10524-WGY, Document 560, Trade Secrets Trial, Transcripts*, 45–46).

Ms Hermle insisted: “Ric Fulop did not breach any fiduciary duty to Markforged because his idea, the one he used to create Desktop metal, was in a completely different space and involved a very different set of technological challenges” (*Desktop Metal Inc. v Markforged Inc., Case 1:18-cv-10524-WGY, Document 560, Trade Secrets Trial, Transcripts*, 47).

Ms Hermle contended: “There will be no evidence that Ric Fulop or Desktop Metal used anything that was confidential or proprietary developed by Markforged” (*Desktop Metal Inc. v Markforged Inc., Case 1:18-cv-10524-WGY, Document 560, Trade Secrets Trial, Transcripts*, 57). Ms Hermle maintained: “Desktop Metal will show you—we will show you all of the extensive work put into developing the innovative metal printers that they've now brought to market and we'll bring in experts in the field to support our trade secrets and damages claims” (*Desktop Metal Inc. v Markforged Inc., Case 1:18-cv-10524-WGY, Document 560, Trade Secrets Trial, Transcripts*, 57).

O'Brien (2018c) suggested that the public dispute posed reputational risks for the two metal 3D printing companies: “The trial had promised to consume money, time and energy for two companies racing to capture a share of an industrial 3D-printing market that is potentially worth billions of dollars per year.” O'Brien (2018c) noted: “Both CEOs were present in the courtroom during opening statements on Tuesday, and Markforged founder and CEO Greg Mark spent hours on the witness stand this week.” O'Brien (2018c) observed: “The trial would have also highlighted plenty of unflattering moments for each company.” Such reputational risks about the public dispute may well have encouraged the parties to reach a settlement.

In a longer piece, O'Brien (2018d) delved into the conflict between Mark and Fulop, Markforged Inc. and Desktop Metal Inc: “The story—as told through court testimony, legal memorandums, and texts and emails included as discovery in the case—provides a rare look inside the usually secretive world of high-growth start-ups, complete with disagreements over intellectual property, key hires and investors, all colored by the personal animosity of two former associates turned competitors.” O'Brien (2018d) commented: “The battle underscores the huge opportunity and enormous sums of money at stake in the field of 3D-printing, which promises to make crucial parts of the manufacturing process much cheaper and faster, potentially changing the way the world makes everything from jet engines to replacement hips. Metal 3D-printing alone could be worth $4 billion per year by 2027, according to market research firm SmarTech Markets Publishing.”

O'Brien (2018d) noted that “others in the Massachusetts 3D-printing industry are surely watching what happens between Markforged and Desktop Metal.” O'Brien (2018d) noted: “Duncan McCallum, the CEO of another Massachusetts metal 3D-printing startup called Digital Alloys Inc., said their competition has important implications for other companies.”

### Settlement

The trade secrets dispute also ended being a stalemate. On the 2nd October 2018, Desktop Metal and Markforged reached a settlement over the claims of breach of trade secrets and confidential information (Jackson, 2018c). The press release stated:

Desktop Metal and Markforged today announced they have reached an agreement that resolves all outstanding litigation between the two companies. Both Desktop Metal and Markforged acknowledge that neither company, nor the individuals named in the litigation, misappropriated any trade secret or confidential information belonging to the other. Further terms and conditions of the settlement will remain confidential (Desktop Metal Inc and Markforged Inc., 2018).

Discussing the settlement, Jackson (2018c) speculated whether there had been a licensing agreement between the two companies: “If this were the case, it wouldn't be the first time this has happened in the 3D printing industry.” She observed that settlements had been reached in other patent infringement disputes in the 3D printing industry: “Formlabs, maker of the Fuse 1 and the Form series of 3D printers, settled an SLA licensing agreement with South Carolina's 3D Systems” (Jackson, 2018c). She noted: “Formlabs was later challenged by EnvisionTEC over similar issues” (Jackson, 2018c). Indeed, Formlabs has also faced action from EnvisionTEC (Biggs, 2014; Long, 2014)–as well as s patent dispute with DWS (Stevenson, 2018). Jackson observed: “While the fine details of Desktop Metal and Markforged have not been disclosed, it would be understandable if the parties involved wished to reach a swift conclusion to the matter—especially as the market for MIM powder-based metal additive systems continues to heat up” (EnivisionTEC, 2016; Jackson, 2018c).

The outcome in the trade secrets dispute between Desktop Metal and Markforged could be contrasted with the much more decisive outcomes of other trade secrets litigation—for instance, Waymo and Google achieved a significant victory against Uber in its settlement over the alleged trade secrets violations by its engineer Anthony Levandowski (Khosrowshahi, 2018). It should be also noted that the dispute between Desktop Metal and Markforged did not escalate into a criminal action over trade secrets—unlike the dispute involving Anthony Levandowski, which resulted in a Federal criminal prosecution for theft of trade secrets (United States Department of Justice, 2020).

However, further litigation between the parties over the settlement suggests that the relationship between the parties were far from “amicable.” Markforged Inc. brought legal action against its rival Desktop Metal, alleging that there had been disparagement in breach of the terms of the settlement.

### Non-disparagement case

The dispute between Markforged Inc. and Desktop Metal Inc. erupted again in 2019, with Markforged alleging that Desktop Metal had breached the settlement through the spreading of allegedly false information (Maffei, 2019). Markforged Inc. brought an action in the United States District Court for the District of Massachusetts *(**Markforged, Inc. v. Desktop Metal, Inc. (1:19-cv-11635) District Court, D. Massachusetts [Non-Disparagement Dispute]**)*.

In 2018, the two companies managed to reach a settlement over their issues, with the court ruling “Both Markforged and Desktop Metal acknowledge that neither company, nor the individuals named in the litigation, misappropriated any trade secret or confidential information belonging to the other.”

In July 2019, Desktop Metal Inc. and Markforged Inc. have renewed their legal battle months after the rival 3D-printing startups signed a settlement over rival claims of trade secrets infringement (Maffei, 2019). Markforged has alleged that Desktop Metal is in breach of contract, and has committed violations of the Lanham Act, and unfair and deceptive acts and practices under Chapter 93A (Davies, 2019). Markforged has sought a jury trial to award three times actual damages to its business, punitive damages, litigation costs and any other relief deemed fit, as well as a permanent injunction against Dekstop Metal, its executives, and its employees. In addition, Markforged desires a settlement based on the US$100K penalty agreement, saying: “Declare that each communication, distribution or dissemination of each false and misleading statement by Desktop Metal about Markforged and its products constitutes a separate occurrence in breach of the parties' Settlement Agreement” (Stevenson, 2019).

In the lawsuit, Markforged alleged: “Notwithstanding the non-disparagement prohibitions in the settlement agreement, Desktop Metal has unleashed yet another scheme to kill Markforged—using dirty tricks against Markforged” (Dowling, 2019). The company argues: “Fulop and his colleagues have acted like proverbial schoolyard bullies, engaging in a persistent pattern of unfair and deceptive conduct, culminating most recently in their dissemination of flagrant falsehoods about Markforged's 3D printers and products” (Dowling, 2019). This is quite strong language. It remains to be seen whether the court approves of the use of such colorful language to describe the dispute.

Markforged has claimed that Desktop Metal has breached the non-disparagement clause in the settlement on a number of occasions, notably in communications with resellers, customers and potential customers (Davies, 2019). In its complaint, Markforged alleged Desktop Metal has breached the prior contract between the two companies in which both parties agreed to cease disparaging each other's businesses and products— “a promise that steadfastly ignored starting even before the ink was dry on the Settlement Agreement” (Jackson, 2019). It also accuses Desktop Metal CEO Ric Fulop of “surreptitiously” incorporating his company while sitting as an active director of the board at Markforged (Jackson, 2019).

In terms of its evidence, Markforged claims that the desktop metal 3D printing company was in breach of its settlement contract when it sent certain marketing materials, or “Battle cards,” to over 100 of its resellers (Boissonneault, 2019). In the flyers, Desktop Metal presents comparisons between its Studio System and the Metal X, which Markforged's lawyers have deemed “false” and a “violation” of their agreement. In the complaint, Markforged was particularly upset about claims made in respect of the safety of the product:

Desktop Metal even went so far as to claim that Markforged's 3D printers and products are unsafe for an office environment and can start a fire because they use “flammable solvents” and Desktop Metal does not—a false statement as Fulop and his fellow bad actors at Desktop Metal well know. To the contrary, Desktop Metal's own data sheet for the solvent used by Desktop Metal reports higher flammability/combustibility characteristics than the solvent used by Markforged (Stevenson, 2019).

As such, the legal team has been sending letters to Desktop Metal, requesting $100 thousand in damages for each reseller and potential customer that has seen the leaflet containing the false statements.

Markforged also accuses Desktop Metal of making false and misleading statements about Markforged's products, “which go well beyond the proverbial rough and tumble” of market competition, including comments allegedly made by Desktop Metal that the Markforged Metal X system “creates a severe contamination risk” and exposes users to toxic solvents and vapors. Markforged is also claiming that, upon being presented with evidence, Desktop Metal “begrudgingly admitted” to making these claims to value add resellers and then “undertook to destroy the marketing materials still in its possession” (Davies, 2019). Markforged accused Desktop Metal of acting like “like proverbial schoolyard bullies,” disseminating false information about the Metal X, and “engaging in a persistent pattern of unfair and deceptive conduct” (Jackson, 2019).

In a statement, Markforged explained the motivation for the litigation:

Metal 3D printing is on pace to change manufacturing as we know it, and Markforged is leading the charge. We believe healthy competition is good for the industry, innovation, and—most importantly—customers. Unfortunately, as alleged in our complaint, Desktop Metal has chosen to compete by spreading false information. Markforged is taking this necessary step to ensure customers are making their buying decisions on facts, not lies (Davies, 2019).

A journalist Stevenson (2019) highlighted the strong language in the complaint: “I have never read a legal claim written with such dramatic flair as this one.” She flagged language such as “Behind Markforged's Back,” “Flagrant Breach,” “Acted Like Proverbial Schoolyard Bullies,” “Flagrant Falsehoods,” “Treacherous And Deceitful Conduct,” “Pure Malevolence,” “Duplicitous Conduct,” and “Inculcated Himself Into The Very Bowels Of Markforged's Business.” It will be interesting whether the court finds such language appropriate for a legal complaint.

In response, Desktop Metal has commented on the dispute: “We are aware of the filing by MarkForged and believe the claims are without merit. We will be addressing the allegations in the appropriate forum” (Davies, 2019). In response to letters contained in the evidence, Desktop Metal's legal representatives said: “That document [the Battle Card] was an internal draft produced in early February 2019” (Stevenson, 2019). They maintained: “To our knowledge, this version of the document was not disseminated by Desktop Metal to any person outside of the Company” (Stevenson, 2019). Nonetheless, they defended the accuracy of the statements: “Desktop Metal does not believe that any of the statements relating to Markforged's products are untrue based on its understanding of those products” (Stevenson, 2019).

Tess Boissonneault observed that “Generally speaking, the additive manufacturing industry is characterized by friendly competition, with many companies continually innovating not only to drive their own products but to bolster and accelerate the AM industry at large” (Boissonneault, 2019). She suggested that this dispute was an exception: “That being said, butting heads is inevitable at times, especially when it comes to issues of intellectual property” (Boissonneault, 2019).

Perhaps the conflict between the two metal 3D printing companies can in part be explained by the commercial interest in the technology. Brian Dowling observed that both companies have received significant commercial funding:

Both companies raised significant amounts of new capital this year. In January, Desktop Metal closed a $160 million Series E funding round led by the venture technology arm of Koch Industries, pushing its total venture haul to $438 million since 2017. In March, Markforged took in $82 million in a Series D funding round led by Boston-based Summit Partners, making its total raised $137 million since 2013 (Dowling, 2019).

This rivalry has amongst other things resulted in intense commercial competition.

A hearing was held in December 2020 and the arbitrator has ruled that Desktop Metal do not owe Markforged any damages associated with the claim. The Markforged Holding Corporation (2021, p. 50) provides this account of the arbitration:

In October 2019, we submitted an Arbitration Demand with JAMS against Desktop Metal alleging breach of the parties' Settlement Agreement pursuant to the non-disparagement obligations therein, as well as a violation of M.G.L. c. 93A. Desktop Metal counterclaimed against us for breach of the parties' Settlement Agreement pursuant to the confidentiality provision therein. The matter proceeded in confidential arbitration and a hearing was held in December 2020. The Arbitration decision was issued on February 26, 2021, and the Arbitrator ruled that neither we nor Desktop Metal were liable pursuant to their respective claims, and that neither party therefore owed any damages to the other.

This further dispute between the parties could be described as another draw or stalemate.

## Further intellectual property litigation

In 2021, there was further patent litigation involving Markforged Inc, and Desktop Metal, being involved in litigation with their competitors and rivals.

### Continuous composites, inc. v. markforged, inc (2021)

In July 2021, Continuous Composites filed a patent infringement lawsuit against Markforged in the United States District Court for the District of Delaware (AP, 2021; Continuous Composites, Inc. v. Markforged, Inc, 2021).

Continuous Composites noted that it was the owner of the patents at issue in this action: U.S. Patent Nos. 9,511,543 (Tyler, 2016); 9,987,798 (Tyler, 2018); 10,744,708 (Tyler, 2020a), and 10,759,109 (Tyler, 2020b). The company argued that Markforged Inc. has infringed this collection of patents:

Markforged manufactures, markets, sells, and uses several 3D printers that use a 3D printing technique Defendant refers to as a Continuous Fiber Reinforcement (CFR) process (the “Accused Products”). The Accused Products extrude a matrix (e.g., Onyx™, Onyx FR™, Onyx FSD™, nylon) in liquid form together with a continuous fiber reinforcement (carbon fiber, Kevlar^®^, HSHT fiberglass, fiberglass) to “3D print” or generate objects, such as industrial parts or rapid prototypes. Examples of the Accused Products include Defendant's Mark Two, Onyx Pro, X5, and X7 printers. The Accused Products are Defendant's flagship products and, on information and belief, are the primary contributors to Defendant's historical revenue (*Continuous Composites, Inc. v. Markforged, Inc, 2021*, Complaint, 4).

Continuous Composites sought remedies in the form of monetary damages for past infringement as well as injunctive relief prohibiting Markforged from continuing to use the technology protected by the Continuous Composites patents.

The Markforged Holding Corporation (2021, p. 50) promised: “We intend to mount a vigorous defense against Continuous Composites in court.” Nonetheless, the Markforged Holding Corporation (2021, p. 50) noted: “We can provide no assurance as to the outcome of any such disputes, and any such actions may result in judgments against us for significant damages.” The Markforged Holding Corporation (2021, p. 50) cautioned: “Resolution of any such matters can be prolonged and costly, and the ultimate results or judgments are uncertain due to the inherent uncertainty in litigation and other proceedings.”

Markforged Inc. put forward a motion for the case to be dismissed. Continuous Composites Inc. has filed a second amended complaint.

In March 2022, Markforged Inc. has filed an answer and counterclaims to the complaint. Markforged Inc. maintained that there was a failure to state a claim: “Continuous Composites fails to plead facts sufficient to show infringement—whether directly, indirectly, literally, or non-literally—of any valid claim of the Asserted Patents or to plead facts sufficient to show any purported infringement was willful or entitles Continuous Composites to enhanced damages” (Markforged Inc., Answer and Counterclaims, 12). Markforged Inc. questions the validity of a number of patents of Continuous Composites Inc., raising issues in respect of inventorship, utility, novelty, non-obviousness, enablement, definiteness, and written description. Markforged Inc. maintains that it has not infringed, induced another to infringe, or contributed to another's infringement of any the patent claims of Continuous Composites Inc.

In terms of remedies, Markforged Inc. calls for a limitation of damages and costs. Markforged Inc. insists that this is not an exceptional case. Markforged Inc. insists that there was no willful infringement. Markforged Inc. says that there should be no injunctive relief. Markforged Inc. invokes various equitable bars to relief. Markforged Inc. notes that there are limitations to patent actions for government sales.

As for its counterclaims, Markforged Inc. has called for declaratory judgment of invalidity of Continuous Composites Inc.'s patents in the dispute. It has also asked for a declaratory judgment of non-infringement of Continuous Composites Inc.'s patents in the dispute. Markforged Inc. has sought a jury trial, asking for a range of remedies in the case.

Markforged Inc. has also asked for an inter partes review of U.S. Patent No. 10,744,708 held by Continuous Composites Inc.

In its 2022 annual report, Markforged Inc. (2022c, Annual Report: F-28) expressed the view about the case: “The Company intends to mount a vigorous defense against Continuous Composites in court.” Markforged Inc. cautioned that “the Company can provide no assurance as to the outcome of any such disputes, and any such actions may result in judgments against Markforged for significant damages.” Markforged Inc. maintained: “The Company does not believe that a loss is probable and did not record a loss contingency for the year ended December 31, 2021.”

### Desktop Metal Inc. v SprintRay (2021)

Likewise, Desktop Metal has also been involved in further intellectual property litigation elsewhere (Desktop Metal Inc. v SprintRay, 2021).

As an early adopter of 3d printing, the field of dentistry has been the subject of a number of pieces of patent litigation—as can be seen in the ClearCorrect litigation in the United States (Rimmer, 2019).

In December 2021, industrial 3D printer manufacturer Desktop Metal was granted a preliminary injunction by a court in Germany that prevents SprintRay from selling its dental systems there (Hanaphy, 2021). Desktop Metal alleged that the technology behind SprintRay's Pro 95 and Pro 55 3D printers infringes upon its patents covering the “layer separation process” of its subsidiary EnvisionTEC. Michael Jafar, CEO of Desktop Health, commented: “We are very happy with the Court's decision” (Hanaphy, 2021). He stressed that the company would vigorously defend its intellectual property: “Desktop Metal's commitment to R&D in hardware, software and material science have resulted in over 650 issued patents and pending patent applications worldwide, which we intend to vigorously enforce” (Hanaphy, 2021). As a result of the ruling, SprintRay is now prohibited from selling, importing, using or storing any product in Germany, which is alleged to violate these patents (Hanaphy, 2021).

The European Patent Office (2020a) has been hosting events in respect of patent law, policy, and practice in respect of 3D printing in the European Union. The European Patent Office (2020b) has also sought to map patent landscapes in respect of 3D printing patents in the European Union. Its report has highlighted how Germany dominates the innovation in additive manufacturing—with six regions among the top fifteen additive manufacturing innovation centers in the European Union.

## Conclusion

This paper has provided a case study of intellectual property conflict over metal 3D printing between two rival United States companies from Boston—Desktop Metal Inc. and Markforged Inc. The dispute raised questions around patent validity, patent infringement, and patent remedies; as well as trade secrets, contract law, consumer law, and unfair competition. The conflict was an inconclusive one—with neither party obtaining advantage from the litigation. It is striking that the judge, jury, and arbitrator took positions in the dispute, which recognized the continuing co-existence of Desktop Metal Inc. and Markforged Inc, enabling future competition and innovation in the metal 3D printing sector. Nonetheless, there will no doubt be further intellectual property litigation in respect of 3D printing in general, and metal 3D printing in particular, given the commercial value associated with the technologies. While there has not been Napster-like litigation in respect of metal 3D printing yet, there could be such issues in the future, especially if the technology goes mainstream (Desai and Magliocca, 2014).

The intensity of the rivalry and feud between Desktop Metal Inc. and Markforged Inc. is startling. Taking a long historical view, White (2001) has argued that rivalry is a key feature of scientific endeavor. He has highlighted the conflicts and competition between scientific figures such as Newton and Leibniz; Lavoisier and Priestley; Darwin and Wallace; Edison and Tesla; the race for the Atom Bomb; Crick and Watson; the space race; and Bill Gates and Larry Ellison. The dispute over intellectual property and metal 3D printing between Greg Mark and Ric Fulop, Markforged Inc. and Desktop Metal Inc. perhaps fits into this pattern of scientific rivalry and feuds. There have certainly been intense patent races in respect of other new technologies—such as HIV/ AIDS diagnostics (Markel, 2020), genomic research in respect of breast and ovarian cancer (Contreras, 2021); and gene-editing CRISPR technologies (Isaacson, 2021). As discussed, the outcome of the dispute between Desktop Metal Inc and Markforged Inc. (2018) is a curious one—with the legal system favoring neither party, and instead recognizing the co-existence of two companies. This could be contrasted with some of the earlier historical patent races, which have clear winners and losers in the legal adjudications.

The field of metal 3D printing still seems to be some way off the place of bounty and plenitude envisaged by Lemley (2015). Technical limitations relating to materials and the technology have continued to create conditions of scarcity. There remain intense and vigorous conflicts over intellectual property and artificial scarcity in the context of metal 3D printing. Thinking about trends in 3D printing and additive manufacturing, Rifkin (2014) envisaged a utopian future of collaborative capitalism. The dispute between Desktop Metal and Markforged would suggest that there is still a culture of competitive capitalism in 3D printing—at least in the field of metal 3D printing. Rifkin's vision of peaceful collaboration and collaboration has not necessarily been realized. Desai (2019) has observed that democratized production poses challenges for regulation. He has highlighted the convergence of technologies—from 3D printing and additive manufacturing to biotechnology and CRISPR gene editing to artificial intelligence and robotics. Desai (2019, p. 251) comments that “such technology forces us to rethink tools of governance and the nature of regulation in the twenty-first century.”

Metal 3D printing has an expanding array of applications and utilities. There has been heavy investment in metal 3D printing by the automotive industry—by companies such as Ford (Chernova, 2018). Rotman (2017) predicted that metal 3D printing “won't replace such century-old production techniques as forging and metal casting, but 3-D printing could create new possibilities in manufacturing—and, just maybe, reimagine the art of metallurgy.” There have been significant application of metal 3d printing in the automotive industry. Likewise, there has been much interest in the use of metal 3D printing in the aerospace industry. There has also been a notable interest in the use of metal 3D printing in maritime industries. There has been an interest of metal 3D printing in respect of consumer goods, the creative industries, and healthcare. Birtchnell and Urry (2016) have investigated whether 3D printing will promote sustainable development.

In 2022, the Biden Administration has sought to accelerate the uptake of metal 3D printing by small-to-medium businesses with its AM Forward policy (White House, 2022). President Joe Biden expressed his personal enthusiasm for 3D Printing and the AM Forward initiative:

3D printing technology—3D printing technology is incredible. It can reduce the parts lead times by as much as 90 percent—not always, but as much as 90 percent—slash material cost by 90 percent, and cut energy use in half. That all helps to lower the cost of making goods here in America. But not all small- and medium-sized firms have access to the resources and financing and support they need to adapt these—to this technology, until today. The executives here today have agreed to launch a new compact between large iconic manufacturers and smaller American suppliers. A commitment by these large companies to help those smaller ones adapt new technologies so we can continue to be the leading exporter of aircrafts and engines and in areas like medical devices, clean energy technologies, and so much more (Biden, 2022).

The White House observed that “not enough American companies are using 3D printing or other high-performance production technologies” (White House, 2022). Under the AM Forward policy, “leading manufacturers will support their U.S.-based suppliers' adoption of new additive capabilities, helping to transform shop floors across the country” (White House, 2022). The Biden Administration has also called on the United States Congress to pass the Bipartisan Innovation Act (White House, 2022). The 3D printing industry has been delighted by this new policy initiative (Hanaphy, 2022). Ric Fulop of Desktop Metal noted: “Additive manufacturing has long held the potential to de-risk supply chains and enable new innovations” (Hanaphy, 2022). He observed: “With manufacturing reshoring already accelerating as a result of the historic supply chain disruption caused by the COVID-19 pandemic, the AM Forward initiative is a timely and progressive approach to modernizing our nation's outdated manufacturing infrastructure with cutting-edge technologies that will help ensure that the work stays here for the long-term” (Hanaphy, 2022). Markforged Inc. (2022b) has also been complimentary about the AM Forward Program.

Representatives of both Desktop Metal Inc. and Markforged Inc. remain upbeat and optimistic about the future of metal 3D printing. In a 2020 interview with 3D Printing Industry, Ric Fulop of Desktop Metal considered trends in 3D printing (Petch, 2020). He predicted: “The next frontier for additive will be in functional end-use applications and mass production” (Petch, 2020). Fulop emphasized: “The industry is now mature enough that we can design machines that actually leverage these technologies into the products that people use every day” (Petch, 2020). He also envisaged: “In this next decade for 3D printing, we are entering an exponential curve because the technology is more affordable, there are more use cases and more supply of raw materials that opens up the application space” (Petch, 2020). Fulop hoped that “[additive manufacturing] will accelerate a greater freedom of product design” (Petch, 2020). Mark Gannon, the Vice President of operations at Markforged, also had his own predictions for 3D printing (Petch, 2020). He expected that “3D printing will continue to permeate the entire factory, evolving from fixtures and tooling to end-use parts” (Petch, 2020). He emphasized that “the industry is sure to realize further uses for the technology—especially as we start to see 3D printed parts pass the industry's most stringent quality and durability certification standards” (Petch, 2020). Gannon predicted: “As the technology matures—through more precise printing technology, and new materials—and leveraging additive becomes more natural as younger engineers already accustomed to the technology join the workforce, we'll see innovation flourish” (2020).

No doubt there will be future conflict over intellectual property and trade in respect of forms of advanced manufacturing—such as metal 3D printing. There will be future competition for such pioneers and trailblazers in metal 3D printing in the field of 3D printing and advanced manufacturing from BRICS/ BASIC nations (Birtchnell et al., 2018).

## Author's note

MR is a Professor in Intellectual Property and Innovation Law at the Faculty of Business and Law in the Queensland University of Technology (QUT). He is the chief investigator in the Australian Research Council Discovery Project, Inventing the Future: Intellectual Property and 3D Printing (2017-2021) (DP 170100758).

## Data availability statement

The original contributions presented in the study are included in the article/supplementary material, further inquiries can be directed to the corresponding author.

## Author contributions

The author confirms being the sole contributor of this work and has approved it for publication.

## Funding

MR is the chief investigator in the Australian Research Council Discovery Project-Inventing the Future: Intellectual Property and 3D Printing (2017–2021) (DP 170100758).

## Conflict of interest

The author declares that the research was conducted in the absence of any commercial or financial relationships that could be construed as a potential conflict of interest.

## Publisher's note

All claims expressed in this article are solely those of the authors and do not necessarily represent those of their affiliated organizations, or those of the publisher, the editors and the reviewers. Any product that may be evaluated in this article, or claim that may be made by its manufacturer, is not guaranteed or endorsed by the publisher.
